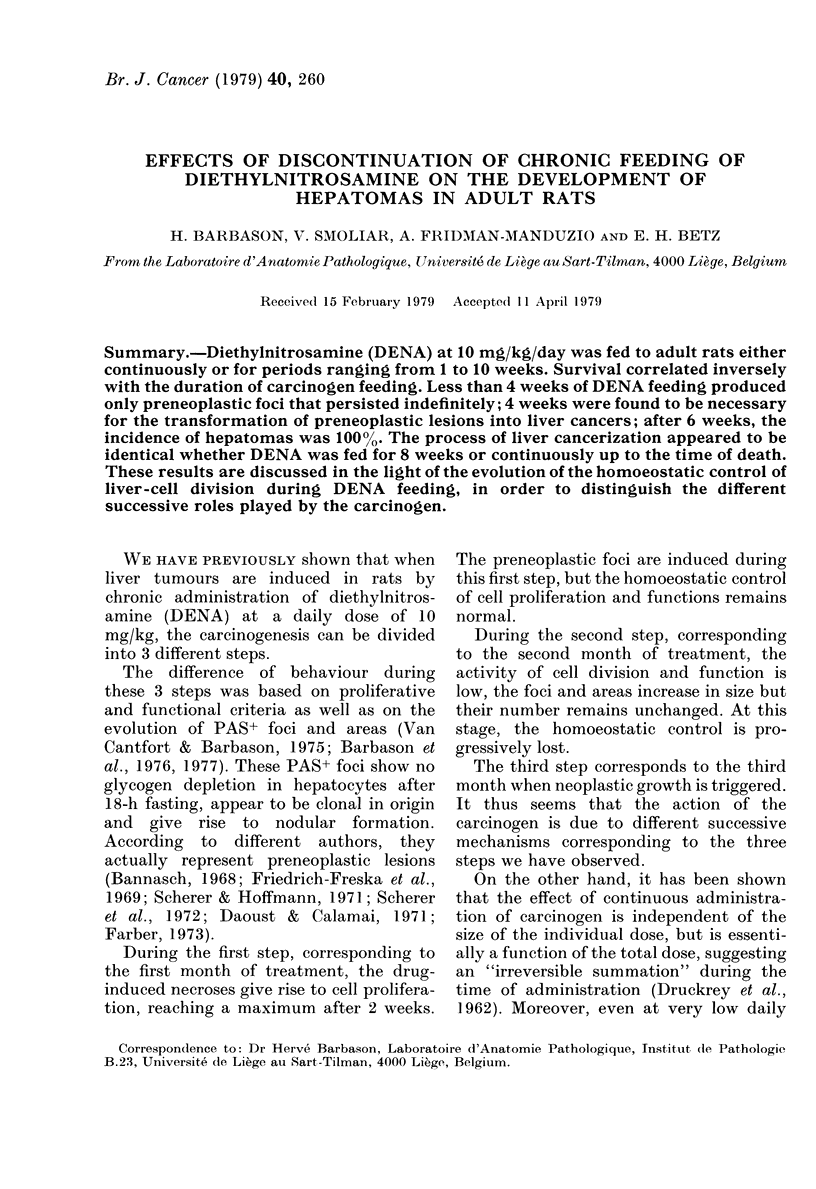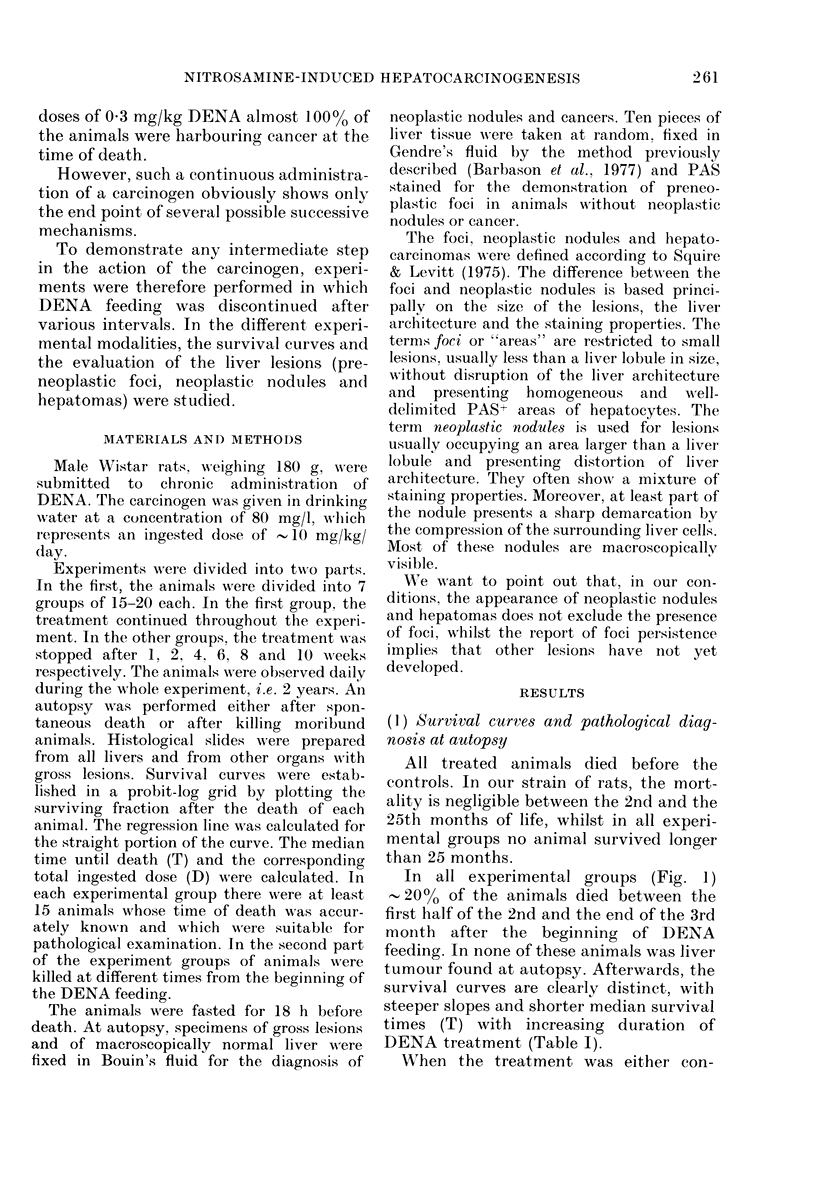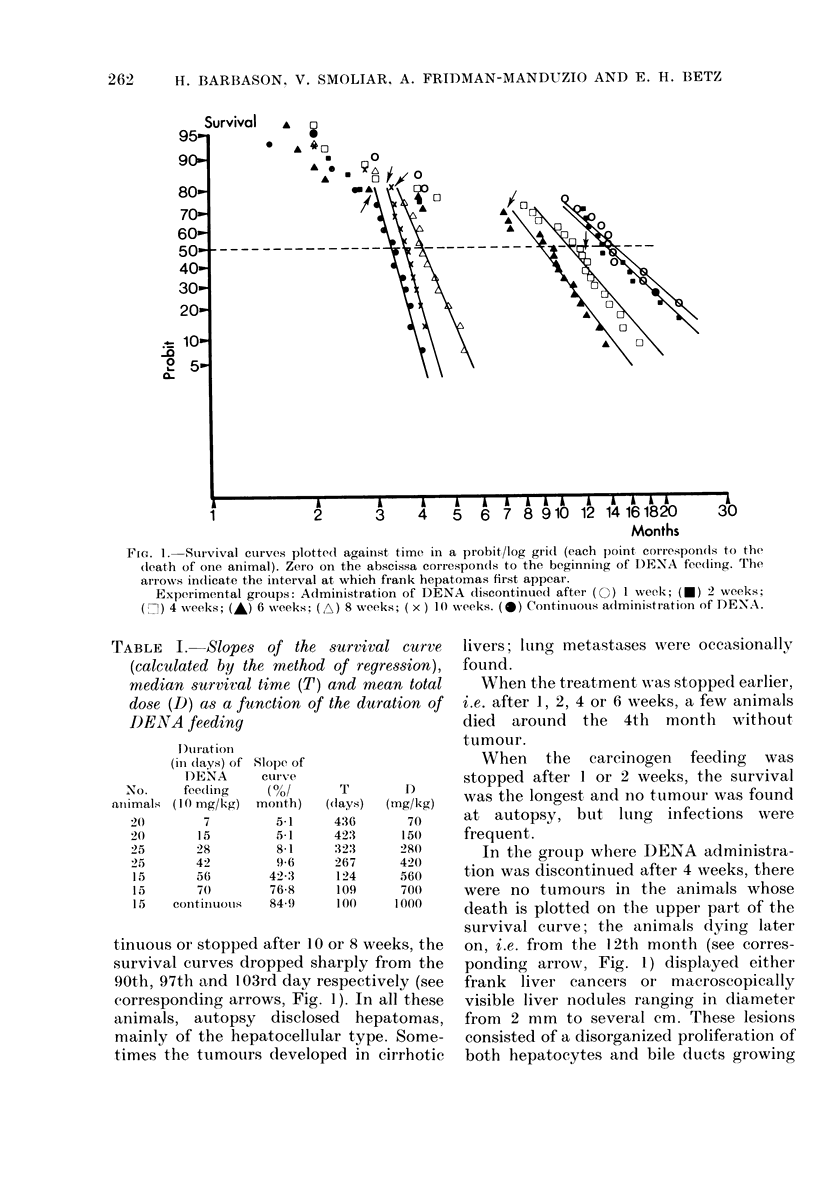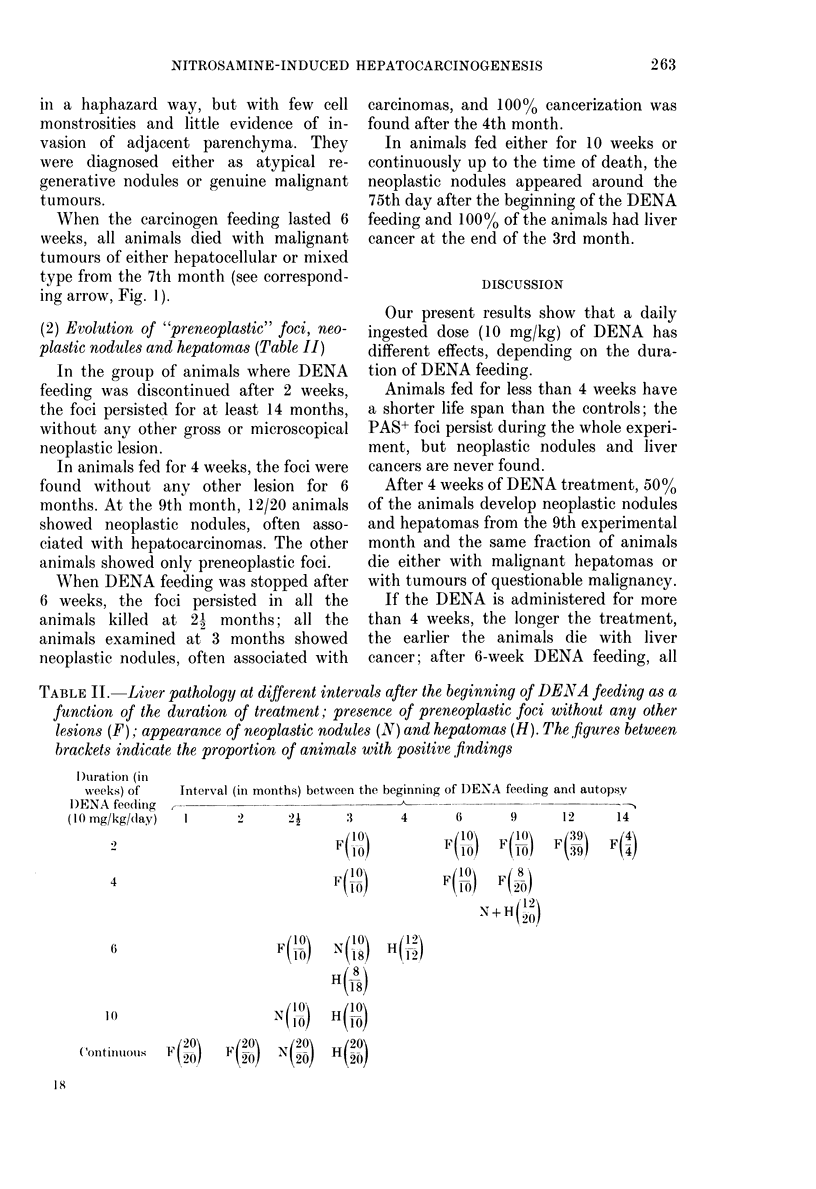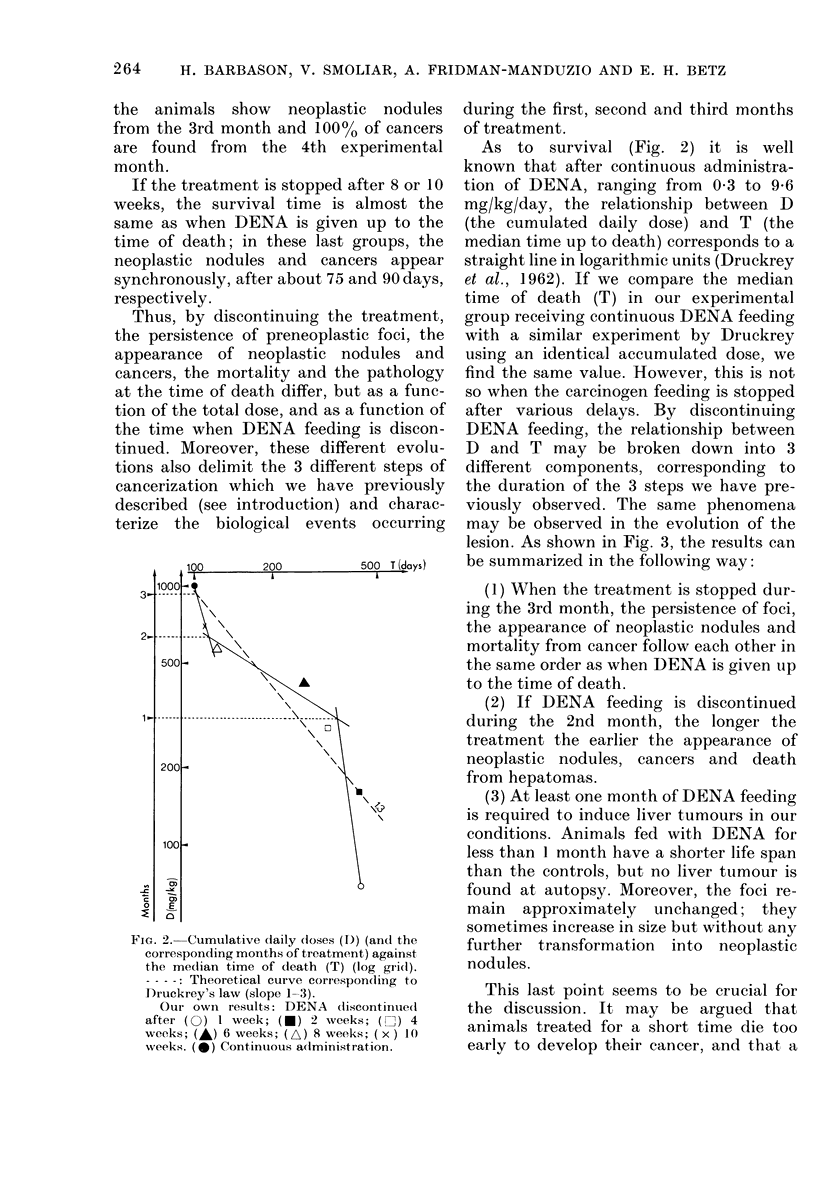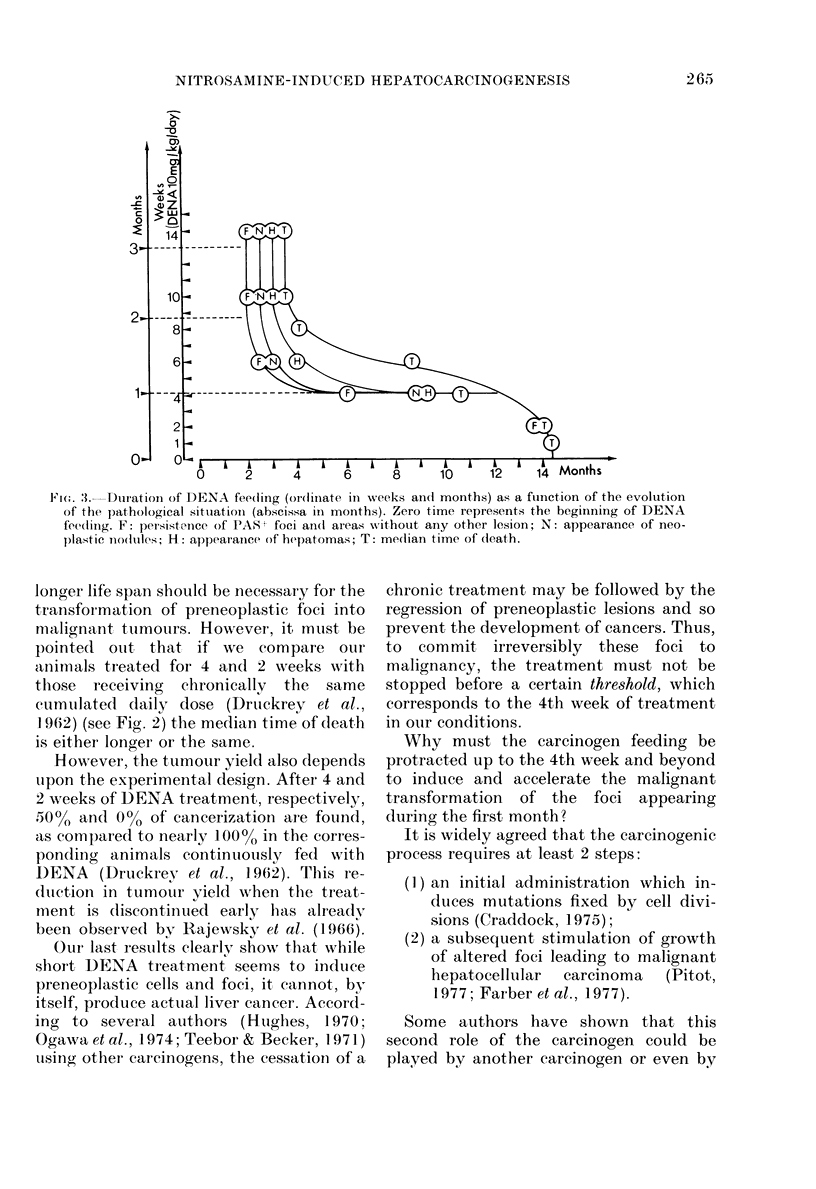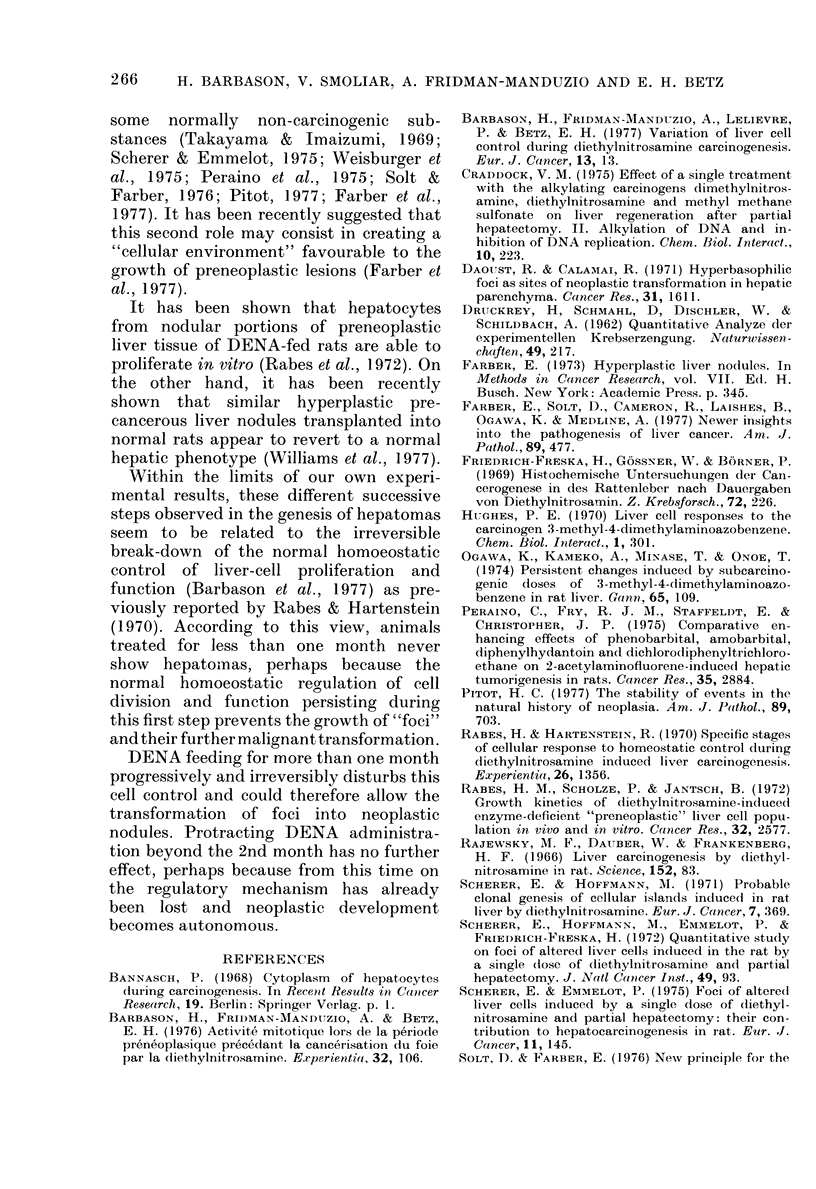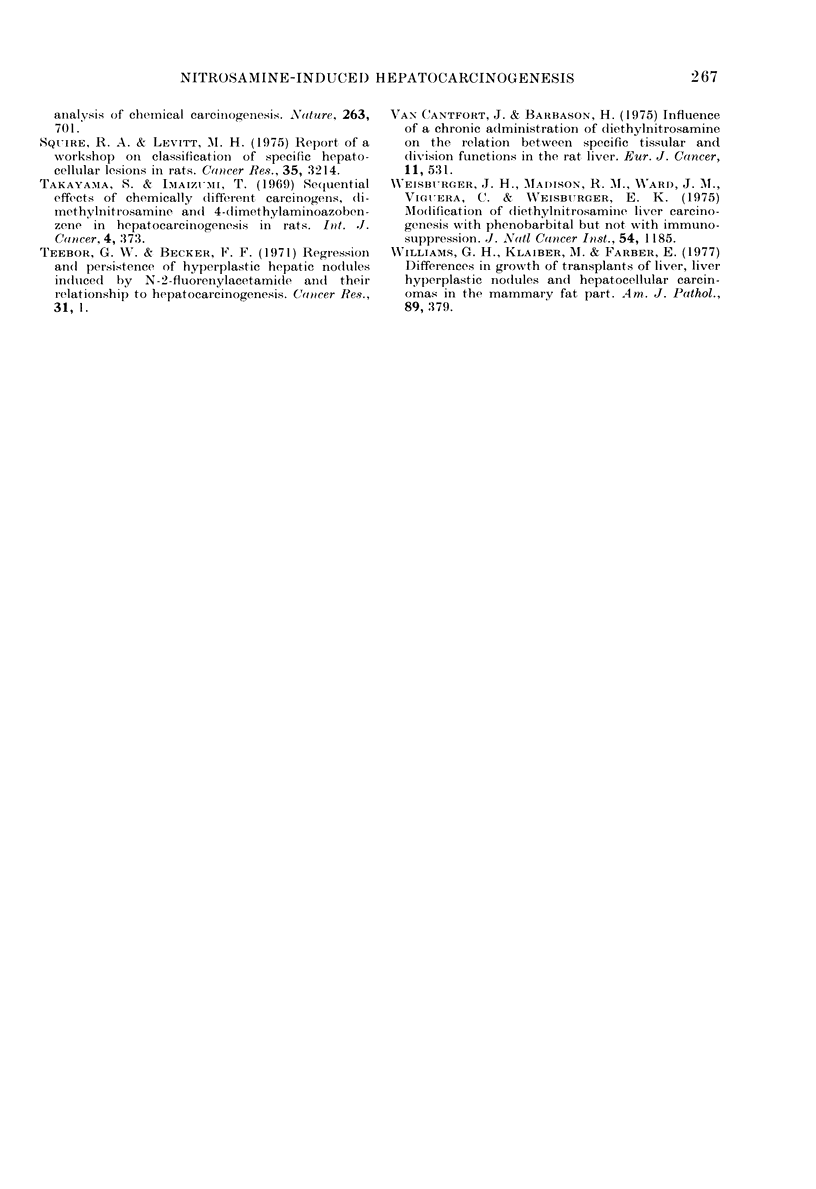# Effects of discontinuation of chronic feeding of diethylnitrosamine on the development of hepatomas in adult rats.

**DOI:** 10.1038/bjc.1979.174

**Published:** 1979-08

**Authors:** H. Barbason, V. Smoliar, A. Fridman-Manduzio, E. H. Betz

## Abstract

Diethylnitrosamine (DENA) at 10 mg/kg was fed to adult rats either continuously or for periods ranging from 1 to 10 weeks. Survival correlated inversely with the duration of carcinogen feeding. Less than 4 weeks of DENA feeding produced only preneoplastic foci that persisted indefinitely; 4 weeks were found to be necessary for the transformation of preneoplastic lesions into liver cancers; after 6 weeks, the incidence of hepatomas was 100%. The process of liver cancerization appeared to be identical whether DENA was fed for 8 weeks or continuously up to the time of death. These results are discussed in the light of the evolution of the homoeostatic control of liver-cell division during DENA feeding, in order to distinguish the different successive roles played by the carcinogen.


*Br. J. Cancer* (1979) **40**, 260

### EFFECTS OF DISCONTINUATION OF CHRONIC FEEDING OF DIETHYLNITROSAMINE ON THE DEVELOPMENT OF HEPATOMAS IN ADULT RATS

H. BARBASON, V. SMOLIAR, A. FRIDMAN-MANDUZIO AND E. H. BETZ

*From the Laboratoire d'Anatomie Pathologique, Université de Liège au Sart-Tilman,* 4000 *Liège, Belgium*

Received 15 February 1979 Accepted 11 April 1979

**Summary.—Diethylnitrosamine (DENA) at 10 mg/kg/day was fed to adult rats either continuously or for periods ranging from 1 to 10 weeks. Survival correlated inversely with the duration of carcinogen feeding. Less than 4 weeks of DENA feeding produced only preneoplastic foci that persisted indefinitely; 4 weeks were found to be necessary for the transformation of preneoplastic lesions into liver cancers; after 6 weeks, the incidence of hepatomas was 100%. The process of liver cancerization appeared to be identical whether DENA was fed for 8 weeks or continuously up to the time of death. These results are discussed in the light of the evolution of the homoeostatic control of liver-cell division during DENA feeding, in order to distinguish the different successive roles played by the carcinogen.**

WE HAVE PREVIOUSLY shown that when liver tumours are induced in rats by chronic administration of diethylnitrosamine (DENA) at a daily dose of 10 mg/kg, the carcinogenesis can be divided into 3 different steps.

The difference of behaviour during these 3 steps was based on proliferative and functional criteria as well as on the evolution of $PAS^+$ foci and areas (Van Cantfort & Barbason, 1975; Barbason *et al.*, 1976, 1977). These $PAS^+$ foci show no glycogen depletion in hepatocytes after 18-h fasting, appear to be clonal in origin and give rise to nodular formation. According to different authors, they actually represent preneoplastic lesions (Bannasch, 1968; Friedrich-Freska *et al.*, 1969; Scherer & Hoffmann, 1971; Scherer *et al.*, 1972; Daoust & Calamai, 1971; Farber, 1973).

During the first step, corresponding to the first month of treatment, the drug-induced necroses give rise to cell proliferation, reaching a maximum after 2 weeks. The preneoplastic foci are induced during this first step, but the homoeostatic control of cell proliferation and functions remains normal.

During the second step, corresponding to the second month of treatment, the activity of cell division and function is low, the foci and areas increase in size but their number remains unchanged. At this stage, the homoeostatic control is progressively lost.

The third step corresponds to the third month when neoplastic growth is triggered. It thus seems that the action of the carcinogen is due to different successive mechanisms corresponding to the three steps we have observed.

On the other hand, it has been shown that the effect of continuous administration of carcinogen is independent of the size of the individual dose, but is essentially a function of the total dose, suggesting an "irreversible summation" during the time of administration (Druckrey *et al.*, 1962). Moreover, even at very low daily

Correspondence to: Dr Hervé Barbason, Laboratoire d'Anatomie Pathologique, Institut de Pathologie B.23, Université de Liège au Sart-Tilman, 4000 Liège, Belgium.

doses of 0·3 mg/kg DENA almost 100% of the animals were harbouring cancer at the time of death.

However, such a continuous administration of a carcinogen obviously shows only the end point of several possible successive mechanisms.

To demonstrate any intermediate step in the action of the carcinogen, experiments were therefore performed in which DENA feeding was discontinued after various intervals. In the different experimental modalities, the survival curves and the evaluation of the liver lesions (preneoplastic foci, neoplastic nodules and hepatomas) were studied.

### MATERIALS AND METHODS

Male Wistar rats, weighing 180 g, were submitted to chronic administration of DENA. The carcinogen was given in drinking water at a concentration of 80 mg/l, which represents an ingested dose of ~10 mg/kg/day.

Experiments were divided into two parts. In the first, the animals were divided into 7 groups of 15–20 each. In the first group, the treatment continued throughout the experiment. In the other groups, the treatment was stopped after 1, 2, 4, 6, 8 and 10 weeks respectively. The animals were observed daily during the whole experiment, *i.e.* 2 years. An autopsy was performed either after spontaneous death or after killing moribund animals. Histological slides were prepared from all livers and from other organs with gross lesions. Survival curves were established in a probit-log grid by plotting the surviving fraction after the death of each animal. The regression line was calculated for the straight portion of the curve. The median time until death (T) and the corresponding total ingested dose (D) were calculated. In each experimental group there were at least 15 animals whose time of death was accurately known and which were suitable for pathological examination. In the second part of the experiment groups of animals were killed at different times from the beginning of the DENA feeding.

The animals were fasted for 18 h before death. At autopsy, specimens of gross lesions and of macroscopically normal liver were fixed in Bouin's fluid for the diagnosis of neoplastic nodules and cancers. Ten pieces of liver tissue were taken at random, fixed in Gendre's fluid by the method previously described (Barbason *et al.*, 1977) and PAS stained for the demonstration of preneoplastic foci in animals without neoplastic nodules or cancer.

The foci, neoplastic nodules and hepatocarcinomas were defined according to Squire & Levitt (1975). The difference between the foci and neoplastic nodules is based principally on the size of the lesions, the liver architecture and the staining properties. The terms *foci* or "areas" are restricted to small lesions, usually less than a liver lobule in size, without disruption of the liver architecture and presenting homogeneous and well-delimited $PAS^+$ areas of hepatocytes. The term *neoplastic nodules* is used for lesions usually occupying an area larger than a liver lobule and presenting distortion of liver architecture. They often show a mixture of staining properties. Moreover, at least part of the nodule presents a sharp demarcation by the compression of the surrounding liver cells. Most of these nodules are macroscopically visible.

We want to point out that, in our conditions, the appearance of neoplastic nodules and hepatomas does not exclude the presence of foci, whilst the report of foci persistence implies that other lesions have not yet developed.

### RESULTS

### (1) *Survival curves and pathological diagnosis at autopsy*

All treated animals died before the controls. In our strain of rats, the mortality is negligible between the 2nd and the 25th months of life, whilst in all experimental groups no animal survived longer than 25 months.

In all experimental groups (Fig. 1) ~20% of the animals died between the first half of the 2nd and the end of the 3rd month after the beginning of DENA feeding. In none of these animals was liver tumour found at autopsy. Afterwards, the survival curves are clearly distinct, with steeper slopes and shorter median survival times (T) with increasing duration of DENA treatment (Table I).

When the treatment was either con-

FIG. 1.—Survival curves plotted against time in a probit/log grid (each point corresponds to the death of one animal). Zero on the abscissa corresponds to the beginning of DENA feeding. The arrows indicate the interval at which frank hepatomas first appear.

Experimental groups: Administration of DENA discontinued after (○) 1 week; (■) 2 weeks; (□) 4 weeks; (▲) 6 weeks; (△) 8 weeks; (×) 10 weeks. (●) Continuous administration of DENA.

TABLE I.—*Slopes of the survival curve (calculated by the method of regression), median survival time (T) and mean total dose (D) as a function of the duration of DENA feeding*

| No. animals | Duration (in days) of DENA feeding (10 mg/kg) | Slope of curve (%/ month) | T (days) | D (mg/kg) |
|---|---|---|---|---|
| 20 | 7 | 5·1 | 436 | 70 |
| 20 | 15 | 5·1 | 423 | 150 |
| 25 | 28 | 8·1 | 323 | 280 |
| 25 | 42 | 9·6 | 267 | 420 |
| 15 | 56 | 42·3 | 124 | 560 |
| 15 | 70 | 76·8 | 109 | 700 |
| 15 | continuous | 84·9 | 100 | 1000 |

tinuous or stopped after 10 or 8 weeks, the survival curves dropped sharply from the 90th, 97th and 103rd day respectively (see corresponding arrows, Fig. 1). In all these animals, autopsy disclosed hepatomas, mainly of the hepatocellular type. Sometimes the tumours developed in cirrhotic livers; lung metastases were occasionally found.

When the treatment was stopped earlier, *i.e.* after 1, 2, 4 or 6 weeks, a few animals died around the 4th month without tumour.

When the carcinogen feeding was stopped after 1 or 2 weeks, the survival was the longest and no tumour was found at autopsy, but lung infections were frequent.

In the group where DENA administration was discontinued after 4 weeks, there were no tumours in the animals whose death is plotted on the upper part of the survival curve; the animals dying later on, *i.e.* from the 12th month (see corresponding arrow, Fig. 1) displayed either frank liver cancers or macroscopically visible liver nodules ranging in diameter from 2 mm to several cm. These lesions consisted of a disorganized proliferation of both hepatocytes and bile ducts growing in a haphazard way, but with few cell monstrosities and little evidence of invasion of adjacent parenchyma. They were diagnosed either as atypical regenerative nodules or genuine malignant tumours.

When the carcinogen feeding lasted 6 weeks, all animals died with malignant tumours of either hepatocellular or mixed type from the 7th month (see corresponding arrow, Fig. 1).

(2) *Evolution of "preneoplastic" foci, neoplastic nodules and hepatomas* (*Table II*)

In the group of animals where DENA feeding was discontinued after 2 weeks, the foci persisted for at least 14 months, without any other gross or microscopical neoplastic lesion.

In animals fed for 4 weeks, the foci were found without any other lesion for 6 months. At the 9th month, 12/20 animals showed neoplastic nodules, often associated with hepatocarcinomas. The other animals showed only preneoplastic foci.

When DENA feeding was stopped after 6 weeks, the foci persisted in all the animals killed at 2½ months; all the animals examined at 3 months showed neoplastic nodules, often associated with carcinomas, and 100% cancerization was found after the 4th month.

In animals fed either for 10 weeks or continuously up to the time of death, the neoplastic nodules appeared around the 75th day after the beginning of the DENA feeding and 100% of the animals had liver cancer at the end of the 3rd month.

DISCUSSION

Our present results show that a daily ingested dose (10 mg/kg) of DENA has different effects, depending on the duration of DENA feeding.

Animals fed for less than 4 weeks have a shorter life span than the controls; the $PAS^+$ foci persist during the whole experiment, but neoplastic nodules and liver cancers are never found.

After 4 weeks of DENA treatment, 50% of the animals develop neoplastic nodules and hepatomas from the 9th experimental month and the same fraction of animals die either with malignant hepatomas or with tumours of questionable malignancy.

If the DENA is administered for more than 4 weeks, the longer the treatment, the earlier the animals die with liver cancer; after 6-week DENA feeding, all

TABLE II.—*Liver pathology at different intervals after the beginning of DENA feeding as a function of the duration of treatment; presence of preneoplastic foci without any other lesions (F); appearance of neoplastic nodules (N) and hepatomas (H). The figures between brackets indicate the proportion of animals with positive findings*

| Duration (in weeks) of DENA feeding (10 mg/kg/day) | Interval (in months) between the beginning of DENA feeding and autopsy | | | | | | | | |
|---|---|---|---|---|---|---|---|---|---|
| | 1 | 2 | 2½ | 3 | 4 | 6 | 9 | 12 | 14 |
| 2 | | | | F(10/10) | | F(10/10) | F(10/10) | F(39/39) | F(4/4) |
| 4 | | | | F(10/10) | | F(10/10) | F(8/20)<br>N+H(12/20) | | |
| 6 | | | F(10/10) | N(10/18)<br>H(8/18) | H(12/12) | | | | |
| 10 | | | N(10/10) | H(10/10) | | | | | |
| Continuous | F(20/20) | F(20/20) | N(20/20) | H(20/20) | | | | | |

the animals show neoplastic nodules from the 3rd month and 100% of cancers are found from the 4th experimental month.

If the treatment is stopped after 8 or 10 weeks, the survival time is almost the same as when DENA is given up to the time of death; in these last groups, the neoplastic nodules and cancers appear synchronously, after about 75 and 90 days, respectively.

Thus, by discontinuing the treatment, the persistence of preneoplastic foci, the appearance of neoplastic nodules and cancers, the mortality and the pathology at the time of death differ, but as a function of the total dose, and as a function of the time when DENA feeding is discontinued. Moreover, these different evolutions also delimit the 3 different steps of cancerization which we have previously described (see introduction) and characterize the biological events occurring

FIG. 2.—Cumulative daily doses (D) (and the corresponding months of treatment) against the median time of death (T) (log grid). - - - -: Theoretical curve corresponding to Druckrey's law (slope 1·3).

Our own results: DENA discontinued after (○) 1 week; (■) 2 weeks; (□) 4 weeks; (▲) 6 weeks; (△) 8 weeks; (×) 10 weeks. (●) Continuous administration.

during the first, second and third months of treatment.

As to survival (Fig. 2) it is well known that after continuous administration of DENA, ranging from 0·3 to 9·6 mg/kg/day, the relationship between D (the cumulated daily dose) and T (the median time up to death) corresponds to a straight line in logarithmic units (Druckrey *et al.*, 1962). If we compare the median time of death (T) in our experimental group receiving continuous DENA feeding with a similar experiment by Druckrey using an identical accumulated dose, we find the same value. However, this is not so when the carcinogen feeding is stopped after various delays. By discontinuing DENA feeding, the relationship between D and T may be broken down into 3 different components, corresponding to the duration of the 3 steps we have previously observed. The same phenomena may be observed in the evolution of the lesion. As shown in Fig. 3, the results can be summarized in the following way:

(1) When the treatment is stopped during the 3rd month, the persistence of foci, the appearance of neoplastic nodules and mortality from cancer follow each other in the same order as when DENA is given up to the time of death.

(2) If DENA feeding is discontinued during the 2nd month, the longer the treatment the earlier the appearance of neoplastic nodules, cancers and death from hepatomas.

(3) At least one month of DENA feeding is required to induce liver tumours in our conditions. Animals fed with DENA for less than 1 month have a shorter life span than the controls, but no liver tumour is found at autopsy. Moreover, the foci remain approximately unchanged; they sometimes increase in size but without any further transformation into neoplastic nodules.

This last point seems to be crucial for the discussion. It may be argued that animals treated for a short time die too early to develop their cancer, and that a

FIG. 3.—Duration of DENA feeding (ordinate in weeks and months) as a function of the evolution of the pathological situation (abscissa in months). Zero time represents the beginning of DENA feeding. F: persistence of PAS+ foci and areas without any other lesion; N: appearance of neoplastic nodules; H: appearance of hepatomas; T: median time of death.

longer life span should be necessary for the transformation of preneoplastic foci into malignant tumours. However, it must be pointed out that if we compare our animals treated for 4 and 2 weeks with those receiving chronically the same cumulated daily dose (Druckrey *et al.*, 1962) (see Fig. 2) the median time of death is either longer or the same.

However, the tumour yield also depends upon the experimental design. After 4 and 2 weeks of DENA treatment, respectively, 50% and 0% of cancerization are found, as compared to nearly 100% in the corresponding animals continuously fed with DENA (Druckrey *et al.*, 1962). This reduction in tumour yield when the treatment is discontinued early has already been observed by Rajewsky *et al.* (1966).

Our last results clearly show that while short DENA treatment seems to induce preneoplastic cells and foci, it cannot, by itself, produce actual liver cancer. According to several authors (Hughes, 1970; Ogawa *et al.*, 1974; Teebor & Becker, 1971) using other carcinogens, the cessation of a chronic treatment may be followed by the regression of preneoplastic lesions and so prevent the development of cancers. Thus, to commit irreversibly these foci to malignancy, the treatment must not be stopped before a certain *threshold*, which corresponds to the 4th week of treatment in our conditions.

Why must the carcinogen feeding be protracted up to the 4th week and beyond to induce and accelerate the malignant transformation of the foci appearing during the first month?

It is widely agreed that the carcinogenic process requires at least 2 steps:

(1) an initial administration which induces mutations fixed by cell divisions (Craddock, 1975);
(2) a subsequent stimulation of growth of altered foci leading to malignant hepatocellular carcinoma (Pitot, 1977; Farber *et al.*, 1977).

Some authors have shown that this second role of the carcinogen could be played by another carcinogen or even by some normally non-carcinogenic substances (Takayama & Imaizumi, 1969; Scherer & Emmelot, 1975; Weisburger *et al.*, 1975; Peraino *et al.*, 1975; Solt & Farber, 1976; Pitot, 1977; Farber *et al.*, 1977). It has been recently suggested that this second role may consist in creating a "cellular environment" favourable to the growth of preneoplastic lesions (Farber *et al.*, 1977).

It has been shown that hepatocytes from nodular portions of preneoplastic liver tissue of DENA-fed rats are able to proliferate *in vitro* (Rabes *et al.*, 1972). On the other hand, it has been recently shown that similar hyperplastic precancerous liver nodules transplanted into normal rats appear to revert to a normal hepatic phenotype (Williams *et al.*, 1977).

Within the limits of our own experimental results, these different successive steps observed in the genesis of hepatomas seem to be related to the irreversible break-down of the normal homoeostatic control of liver-cell proliferation and function (Barbason *et al.*, 1977) as previously reported by Rabes & Hartenstein (1970). According to this view, animals treated for less than one month never show hepatomas, perhaps because the normal homoeostatic regulation of cell division and function persisting during this first step prevents the growth of "foci" and their further malignant transformation.

DENA feeding for more than one month progressively and irreversibly disturbs this cell control and could therefore allow the transformation of foci into neoplastic nodules. Protracting DENA administration beyond the 2nd month has no further effect, perhaps because from this time on the regulatory mechanism has already been lost and neoplastic development becomes autonomous.

### REFERENCES

BANNASCH, P. (1968) Cytoplasm of hepatocytes during carcinogenesis. In *Recent Results in Cancer Research*, **19**. Berlin: Springer Verlag. p. 1.

BARBASON, H., FRIDMAN-MANDUZIO, A. & BETZ, E. H. (1976) Activité mitotique lors de la période prénéoplasique précédant la cancérisation du foie par la diethylnitrosamine. *Experientia*, **32**, 106.

BARBASON, H., FRIDMAN-MANDUZIO, A., LELIEVRE, P. & BETZ, E. H. (1977) Variation of liver cell control during diethylnitrosamine carcinogenesis. *Eur. J. Cancer*, **13**, 13.

CRADDOCK, V. M. (1975) Effect of a single treatment with the alkylating carcinogens dimethylnitrosamine, diethylnitrosamine and methyl methane sulfonate on liver regeneration after partial hepatectomy. II. Alkylation of DNA and inhibition of DNA replication. *Chem. Biol. Interact.*, **10**, 223.

DAOUST, R. & CALAMAI, R. (1971) Hyperbasophilic foci as sites of neoplastic transformation in hepatic parenchyma. *Cancer Res.*, **31**, 1611.

DRUCKREY, H, SCHMAHL, D, DISCHLER, W. & SCHILDBACH, A. (1962) Quantitative Analyze der experimentellen Krebserzengung. *Naturwissenchaften*, **49**, 217.

FARBER, E. (1973) Hyperplastic liver nodules. In *Methods in Cancer Research*, vol. VII. Ed. H. Busch. New York: Academic Press. p. 345.

FARBER, E., SOLT, D., CAMERON, R., LAISHES, B., OGAWA, K. & MEDLINE, A. (1977) Newer insights into the pathogenesis of liver cancer. *Am. J. Pathol.*, **89**, 477.

FRIEDRICH-FRESKA, H., GÖSSNER, W. & BÖRNER, P. (1969) Histochemische Untersuchungen der Cancerogenese in des Rattenleber nach Dauergaben von Diethylnitrosamin. *Z. Krebsforsch.*, **72**, 226.

HUGHES, P. E. (1970) Liver cell responses to the carcinogen 3-methyl-4-dimethylaminoazobenzene. *Chem. Biol. Interact.*, **1**, 301.

OGAWA, K., KAMEKO, A., MINASE, T. & ONOE, T. (1974) Persistent changes induced by subcarcinogenic doses of 3-methyl-4-dimethylaminoazobenzene in rat liver. *Gann*, **65**, 109.

PERAINO, C., FRY, R. J. M., STAFFELDT, E. & CHRISTOPHER, J. P. (1975) Comparative enhancing effects of phenobarbital, amobarbital, diphenylhydantoin and dichlorodiphenyltrichloroethane on 2-acetylaminofluorene-induced hepatic tumorigenesis in rats. *Cancer Res.*, **35**, 2884.

PITOT, H. C. (1977) The stability of events in the natural history of neoplasia. *Am. J. Pathol.*, **89**, 703.

RABES, H. & HARTENSTEIN, R. (1970) Specific stages of cellular response to homeostatic control during diethylnitrosamine induced liver carcinogenesis. *Experientia*, **26**, 1356.

RABES, H. M., SCHOLZE, P. & JANTSCH, B. (1972) Growth kinetics of diethylnitrosamine-induced enzyme-deficient "preneoplastic" liver cell population *in vivo* and *in vitro*. *Cancer Res.*, **32**, 2577.

RAJEWSKY, M. F., DAUBER, W. & FRANKENBERG, H. F. (1966) Liver carcinogenesis by diethylnitrosamine in rat. *Science*, **152**, 83.

SCHERER, E. & HOFFMANN, M. (1971) Probable clonal genesis of cellular islands induced in rat liver by diethylnitrosamine. *Eur. J. Cancer*, **7**, 369.

SCHERER, E., HOFFMANN, M., EMMELOT, P. & FRIEDRICH-FRESKA, H. (1972) Quantitative study on foci of altered liver cells induced in the rat by a single dose of diethylnitrosamine and partial hepatectomy. *J. Natl Cancer Inst.*, **49**, 93.

SCHERER, E. & EMMELOT, P. (1975) Foci of altered liver cells induced by a single dose of diethylnitrosamine and partial hepatectomy: their contribution to hepatocarcinogenesis in rat. *Eur. J. Cancer*, **11**, 145.

SOLT, D. & FARBER, E. (1976) New principle for the analysis of chemical carcinogenesis. *Nature*, **263**, 701.

SQUIRE, R. A. & LEVITT, M. H. (1975) Report of a workshop on classification of specific hepatocellular lesions in rats. *Cancer Res.*, **35**, 3214.

TAKAYAMA, S. & IMAIZUMI, T. (1969) Sequential effects of chemically different carcinogens, dimethylnitrosamine and 4-dimethylaminoazobenzene in hepatocarcinogenesis in rats. *Int. J. Cancer*, **4**, 373.

TEEBOR, G. W. & BECKER, F. F. (1971) Regression and persistence of hyperplastic hepatic nodules induced by N-2-fluorenylacetamide and their relationship to hepatocarcinogenesis. *Cancer Res.*, **31**, 1.

VAN CANTFORT, J. & BARBASON, H. (1975) Influence of a chronic administration of diethylnitrosamine on the relation between specific tissular and division functions in the rat liver. *Eur. J. Cancer*, **11**, 531.

WEISBURGER, J. H., MADISON, R. M., WARD, J. M., VIGUERA, C. & WEISBURGER, E. K. (1975) Modification of diethylnitrosamine liver carcinogenesis with phenobarbital but not with immunosuppression. *J. Natl Cancer Inst.*, **54**, 1185.

WILLIAMS, G. H., KLAIBER, M. & FARBER, E. (1977) Differences in growth of transplants of liver, liver hyperplastic nodules and hepatocellular carcinomas in the mammary fat part. *Am. J. Pathol.*, **89**, 379.